# Ultra-Long-Chain Sorbitol Esters Tailoring Thermo-Responsive Rheological Properties of Oleogels

**DOI:** 10.3390/foods14061030

**Published:** 2025-03-18

**Authors:** Marcelo Gomes Soares, Paula Kiyomi Okuro, Marcos Fellipe da Silva, Rosana Goldbeck, Rosiane Lopes Cunha

**Affiliations:** Department of Food Engineering and Technology (DETA), School of Food Engineering (FEA), University of Campinas (UNICAMP), Campinas 13083-862, SP, Brazil; marcelosoares1997@hotmail.com (M.G.S.); paulaokuro@gmail.com (P.K.O.); marcosfellipe96@hotmail.com (M.F.d.S.); goldbeck@unicamp.br (R.G.)

**Keywords:** oleogelator, saturated fat, transesterification, esters, rheology

## Abstract

Oleogels must replicate the rheological behavior of saturated fats at processing and consumption temperatures to maintain their physical stability and sensory acceptance. Thus, multicomponent oleogels present a promising approach since oleogelators can exhibit structuring and melting at different temperatures. The aim of the study was to produce a mixture of ultra-chain-long esters capable of structuring and modulating rheological behavior in response to temperature exposure. Therefore, enzymatic transesterification between sorbitol and fully hydrogenated crambe oil (FHCO) was performed to produce a mixture of ultra-long-chain sorbitan esters (SB) for efficient structuring of sunflower oil. SB generated in a reaction medium consisting exclusively of ethanol (60 °C, 200 rpm, 1:1 molar ratio) was selected for its high sorbitol consumption (~95%). While SB oleogels exhibited higher gel strength at 5 °C, at 25 °C, FHCO oleogels were stiffer, showing the gradual melting of SB oleogels evaluated by temperature-dependent rheological analyses and thermal properties. Oleogelation inhibited hydroperoxide formation compared to sunflower oil over 30 days. Results highlight the potential of multicomponent oleogels based on ultralong-chain esters for healthier and more stable high-lipid products. Modulating rheological thermoresponsiveness ensures physical stability under refrigeration while providing a texture similar to saturated fats during spreading and swallowing.

## 1. Introduction

Oleogels are formed by the physical immobilization of liquid oil in a three-dimensional network, generating a semi-solid structure [1]. As they are predominantly composed of oils containing mostly polyunsaturated fatty acids, oleogels are one of the most promising alternatives for replacing saturated fats in food products [2]. Reducing saturated fat intake is recommended by the World Health Organization (WHO) due to its direct association with obesity and an increased risk of cardiovascular diseases, responsible for 32% of all deaths worldwide [3]. Thus, the use of oleogels not only aligns with public health recommendations but also promotes the development of healthier and more innovative food products.

Although oleogels provide nutritional benefits, achieving consumer sensory acceptance requires that these structures mimic the technological properties of saturated fats, such as physical stability, textural attributes, and melting sensations in the mouth during oral processing [4]. In this sense, the rheological response of oleogels under different temperature conditions during storage and consumption becomes a crucial aspect to be investigated. As potential substitutes for saturated fats, oleogels can be used in butter analogs, margarine, ice cream, cheese, processed meats, baked goods, and other products [5]. While many of these products require refrigeration temperatures (4–7 °C) during storage, it is essential that oleogels have a melting profile capable of providing the desired functionality at higher temperatures, such as spreadability (10–25 °C) and oral conditions (37 °C) [6]. However, there is still a lack of oleogels that combine these characteristics [7], due to the limited amount of commercially available oleogelators.

A number of oleogelators have been investigated in food structures, with ethylcellulose and plant waxes being particularly notable. Although ethylcellulose exhibits excellent oil-structuring capabilities, it is impractical to reach the high temperature (~140 °C) required to dissolve it in oil during large-scale operations [8]. Similarly, although wax-based oleogels provide hedonic characteristics similar to saturated vegetable fats, stability issues over long periods and residual flavor limit their application in food products [9]. This highlights the need for other kinds of oleogelator systems capable of overcoming such challenges. In this context, emulsifiers such as carbohydrate-based esters could be used as an alternative, since they are amphiphilic compounds considered safe for humans and the environment. Although these compounds have been widely studied as emulsifiers (e.g., Tweens and Spans), they are less explored in the field of oleogels [10].

Among the available carbohydrate esters, sorbitol-derived esters can exert influence on the rheological behavior of oleogels. However, a concentration of at least 18% (*w*/*w*) of sorbitan monostearate is required to form a three-dimensional network, making their industrial application unfeasible [11]. Given the limited oleogel-forming capacity of commercially available sorbitan esters, some studies have investigated the potential of using this carbohydrate ester in combination with other oleogelators. Furthermore, although it is known that the gelling capacity increases linearly with the length of their carbon chains (C10 to C31) [12], to the best of our knowledge, no comprehensive and extensive investigation has been conducted on the production of sorbitan esters with ultra-long fatty acid chains and their exploration as oleogelators.

Fully hydrogenated crambe oil (*Crambe abyssinica* Hochst) (FHCO) stands out as a promising donor of the hydrophobic portion for sorbitol ester production. FHCO is a hard fat composed of 56% behenic acid (C22:0), 31.6% stearic acid (C18:0), 6.3% arachidic acid (C20:4), 2.8% palmitic acid (C16:0), and other fatty acids [13]. Recent industrial interest in FHCO has been driven by the presence of behenic acid as its main component. Rarely found in natural sources, behenic acid has significant potential in the development of low-calorie fats due to its low bioavailability and absorption, as well as its excellent structuring capacity [14,15]. Behenic acid is a product of the hydrogenation of erucic acid found in crambe oil, making FHCO a product without consumption restrictions [16]. Due to its six hydroxyl groups, sorbitol can be esterified with the different fatty acids available in FHCO, producing a mixture of esters that can be advantageous for the formation of oleogels.

In this context, as the main objective of this study, a new multicomponent oleogelator composed of a mixture of sorbitan esters was produced using a more sustainable route. For the synthesis of the ester mixture named sorbitan behenate (SB), FHCO was selected as the donor of the hydrophobic portion, while sorbitol served as the donor of the hydrophilic fraction. This sorbitol ester mixture aimed to reproduce the rheological behavior of saturated fats under temperature variations. This strategy opens new opportunities to enable the application of oleogels in high-lipid products on an industrial scale, providing techno-functionality and texture perception (melting) more similar to saturated fats under different processing and consumption conditions.

## 2. Materials and Methods

### 2.1. Material

D-sorbitol and ethanol were obtained from Dinâmica Química (Brazil). Lipase immobilized on acrylic resin (≥5000 U g^−1^, recombinant, expressed in *Aspergillus niger*), also known as *Candida antarctica* lipase B (CALB), was purchased from Sigma-Aldrich (Sigma Aldrich, Norfolk, UK). Fully hydrogenated crambe oil (FHCO) was supplied by Cargill Agricola S/A. Sorbitan monostearate was purchased from Sigma Aldrich (50% purity; Sigma Aldrich, Norfolk, UK). Sunflower oil (SFO) (Bunge Alimentos S.A., Gaspar, Brazil) was purchased from the local market. Other chemicals were of analytical grade and used without the need for further purification.

### 2.2. Screening Reactions

#### 2.2.1. Hydrolytic and Transesterification Activities of CALB

CALB activity was determined by the hydrolysis of *p*-nitrophenyl laurate (*p*-NPL) in a mixture containing lauric acid and *p*-nitrophenol [17]. The reaction was initiated by adding 10 μL of *p*-NPL to the mixture composed of 20 mg of CALB and 990 μL of 50 mM phosphate buffer at pH 7.0. The reaction mixture was incubated at 60 °C for 10 min. Subsequently, 250 μL of the resulting supernatant was extracted and combined with 1 mL of 0.1 M NaOH. The resulting mixture was filtered, and its absorbance was measured at 410 nm using a visible light spectrophotometer (Thermo Scientific—Model Genesys 20, Waltham, MA, USA). For the purposes of this study, one enzymatic hydrolysis unit (U) was defined as the amount of lipase capable of hydrolyzing 1 μmol of *p*-NPL per minute under the specified reaction.

The activity of CALB was assessed by the transesterification reaction between *p*-NPL and ethanol according to [18] with some modifications. First, the mixture containing 500 μL of *p*-NPL (20 mM in n-heptane) and 20 mg of CALB was prepared in 2 mL Eppendorf tubes. Then, the reaction was started by adding 30 μL of ethanol to the mixture. Reactions were carried out at 60 °C for 30 min. Subsequently, 25 μL of the supernatant were collected and mixed with 1 mL of NaOH (0.1 M) in the cuvette. Released *p*-NP was determined at 410 nm against a blank without enzyme using a spectrophotometer (Thermo Scientific—Model Genesys 20, Waltham, MA, USA). The unit of enzymatic synthesis activity (U) was defined as the amount of lipase releasing 1 μmol of *p*-NP per minute under the reaction conditions. Standard *p*-NP curves were prepared in the concentration range of 0 to 0.035 μmol. mL^−1^.

#### 2.2.2. Plackett-Burman (PB) Experimental Design

An experimental design consisting of eleven experimental runs, including 3 central points, was generated (Protimiza Experimental Design—version 19, Campinas, Brazil) with a significance level of 95%. This design aimed to investigate the effects of four parameters: temperature, sorbitol-FHCO molar ratio, agitation speed, and ethanol-water ratio on the reaction medium. Each parameter was tested at two levels called low (−1) and high (+1). Selected ranges for the components were as follows: 40 (−1)–60 (+1) °C for temperature, 1:1 (−1)–1:5 (+1) for FHCO-sorbitol molar ratio, 200 (−1)–500 (+1) rpm for agitation speed, and 100:0 (−1)–50:50 (+1) (*v*/*v*) for ethanol-water ratio. These conditions were evaluated based on the existing literature on the production of carbohydrate esters [19,20,21,22,23]. The independent and dependent variables studied in PB are presented in Table 1.

Experiments were conducted by combining sorbitol, FHCO, CALB, and a volume of 15 mL of reaction medium (with a concentration of 1 g of CALB per 75 mL of reaction medium) in 50 mL Falcon tubes over 48 h. Quantification of unreacted sorbitol was performed using a high-performance liquid chromatography (HPLC) system (Thermo Fisher Scientific Inc., Accela, USA) equipped with a refractive index detector (Thermo Fisher Scientific Inc., Accela, Waltham, MA, USA).

The methodology, adapted from [24], was performed using elution on an Agilent Hi-Plex Ca column at 85 °C with ultrapure water as the mobile phase at a flow rate of 0.6 mL min^−1^. The conversion of substrates to esters (*CTE*) was estimated using Equation (1).

(1) $$CTE\;\left(\%\right)=\left(\frac{C_{si}-C_{sf}}{C_{si}}\right)\times 100$$

where *C_si_* represents the initial sorbitol concentration before the reaction, while *C_sf_* represents the final sorbitol concentration after the reaction.

The experimental condition that demonstrated the highest consumption was selected for subsequent experiments. After the reaction, the CALB was filtered for reusability tests. The filtrate was subjected to a drying process in a forced circulation oven at a temperature of 80 ± 1 °C for a period of 12 h. For reuse studies, after the end of each transesterification step, CALB was recovered from the filtration process, thereby allowing its use in the subsequent cycle.

#### 2.2.3. Fourier Transform Infrared Spectroscopy (FTIR)

FTIR spectra were acquired for SB and FHCO using an IRPrestige-21 Fourier transform infrared spectrophotometer manufactured by Shimadzu (Kyoto, Japan). This analysis was carried out with the aim of qualitatively confirming the presence of ester groups in the SB structure. Spectral measurements covered the range from 400 cm^−1^ to 4000 cm^−1^, using a resolution of 4 cm^−1^ and accumulating 16 scans per sample. FTIR measurements were performed on potassium bromide (KBr) disks, with a sample-to-KBr ratio of 1 mg to 100 mg [25].

### 2.3. Evaluation of SB Oil-Structuring Capacity

#### Preparation of Oleogels

Oleogelation capacity was evaluated by mixing SB (10% *w*/*w*) with SFO. In order to obtain a clear solution, heating (90 ± 2 °C) and magnetic stirring at 300 rpm (Heidolph Instruments, Schwabach, Germany) were employed for 30 min. After this period of time, the samples were immediately transferred to an ice bath and allowed to cool for 30 min. After cooling, the samples were stored at 5 ± 1 °C or 25 ± 1 °C (refrigerator or room temperature, respectively) for at least 48 h before characterization. As a control, oleogels using FHCO and SM as oleogelators were prepared under the same conditions as SB, aiming to examine the influence of the hydrophilic moiety and lipophilic chain length on the structural properties of the oleogels [26].

### 2.4. Characterization of the Oleogels

#### 2.4.1. Rheological Measurements

All rheological measurements were carried out using a stress-controlled rheometer Physica MCR 301 (Anton Paar, Graz, Austria) equipped with a Peltier system. Sandblasted parallel-plate geometry (Ø = 50 mm) with a roughness of 5–7 μm and a gap of 500 μm was used.

- Frequency and strain sweeps (isothermal measurements)

Strain sweeps were performed between 0.01 and 100% to identify the linear viscoelastic region (LVR). Frequency sweeps (0.01–10 Hz) were performed with a strain value within the linear viscoelastic region of the oleogels. The frequency-dependent behavior of the oleogels was assessed by recording the storage or elastic (G′) and loss or viscous (G″) moduli as a function of frequency. Frequency and strain measurements were performed at 5 and 25 °C [27].

- Temperature sweeps (non-isothermal measurements)

The behavior of elastic (G′) and viscous (G″) moduli was investigated during the cooling and heating cycles, disclosing sol-gel and gel-sol transitions, respectively. Temperature sweeps were performed simulating the formation of a gel network under cooling at a rate with a fixed frequency (1 Hz) and strain value defined within LVR. Initially, the equipment was preheated to 90 °C, and then the samples were left isothermally for 1 min, cooled from 90 to 5 °C at a rate of 5 °C.min^−1^, held isothermally for 1 min, and heated again to 90 °C at a rate of 5 °C.min^−1^. The crossover temperature (G′ = G″) during cooling was defined as the gelation temperature (T_sol-gel_), and the crossover temperature during heating was considered as the gel-sol transition temperature (T_gel-sol_) [27].

#### 2.4.2. Differential Scanning Calorimetry (DSC)

The thermal properties of the oleogels were assessed using a differential scanning calorimeter (TA Instruments DSC-2920 model, New Castle, DE, USA), equipped with a mechanical cooling system. An aluminum pan containing the sample (5 to 7 mg) and an empty pan used as a reference were placed in the equipment. The samples were initially equilibrated at 25 °C, heated to 90 °C (heating stage I), held under isothermal conditions for 1 min, then cooled to 5 °C (cooling stage), held under isothermal conditions for 1 min, and finally heated again to 90 °C (heating stage II). Heating and cooling ramps were performed at a constant rate of 5 °C.min^−1^. Data were processed using TA Universal Analysis software 5.5 (TA Instruments, New Castle, DE, USA) to evaluate the following thermal properties: crystallization onset temperature (T_C1, onset_) associated with the first crystallization peak, melting (ΔH_m_), and crystallization (ΔH_c_) enthalpy [28].

#### 2.4.3. Polarized Light Microscopy (PLM)

The crystal morphology of the oleogels was examined using a polarized light microscope (Olympus System Microscope model BX 50, Olympus America Inc., Center Valley, PA, USA) equipped with a digital camera (Nikon DS-Ri1, Melville, NY, USA). Images were analyzed using NIS-Elements Microscope Image Software (Nikon, Melville, NY, USA). A small amount of oleogel was placed on a glass slide and carefully covered with a cover slip. To simulate oleogel preparation conditions, the following procedure was applied: (i) the sample was heated to 90 °C using a hot stage connected to a Linkam T95 System Controller (Linkam Scientific Instrument Ltd., Surrey, UK); (ii) isothermal conditions were kept for 1 min; (iii) subsequent cooling to 5 °C; (iv) isothermal conditions maintained for another 1 min; and (v) finally, the glass slides were stored at 5 °C for 48 h prior to image capture.

Heating and cooling ramps were conducted at a constant rate of 5 °C.min^−1^. Polarized microscopy images were analyzed with the public domain software ImageJ (https://imagej.net/ij/) to determine the fractal dimension of the flocs, according to the methodology of [29]. The images of FHCO, SB, and SM oleogels were analyzed. The box-counting fractal dimension (FD) of the micrographs was determined using the software ImageJ and the FracLac v2.5-1d plug-in for ImageJ (https://imagej.net/ij/plugins/fraclac/fraclac.html, accessed on 12 March 2025). The micrographs were transformed into 8-bit grayscale images of 404–303 pixels and then thresholded.

#### 2.4.4. Oil Binding Capacity (OBC)

Oil binding capacity was assessed using the methodology described by [30] with minor modifications. Approximately 1 g of freshly prepared oleogel was carefully weighed and transferred into 1.5 mL tubes. Subsequently, the oleogels were stored at either 5 or 25 °C for 48 h before centrifu-gation. After 48 h, the accelerated stability test was performed by subjecting the oleogel samples to centrifugation at 14,000 rpm (21.8 × 10^3^ g) for 30 min using a microcentrifuge (5418 R Eppendorf, Hamburg, Germany). Then, the released oil was drained using qualitative filter paper (Whatman #1) by inverting the tubes for 30 min. The OBC percentage was calculated using Equation (2) [31].

(2) $$OBC\%=\left[1-\frac{{(m}_{i}-m_{f})}{m_{i}}\right]\times 100$$

where m_i_ is the weight of the sample before centrifugation and m_f_ is the weight after the oil drainage.

#### 2.4.5. Oxidative Stability (OS)

The oxidative stability (OS) of the oleogels was assessed by determining the peroxide value (PV) [32]) at room temperature (25 °C) with exposure to light and air for 30 days. Measurements were performed at 0, 7, 15, and 30 days of storage. As a control, SFO (without oleogelator) was heated for 30 min at 90 ± 2 °C under magnetic stirring at 300 rpm (Heidolph Instruments, Schwabach, Germany) and subsequently cooled in an ice bath for 30 min to replicate the oleogel preparation conditions. This sample was named SFO. Oleogels were heated to 70 °C until complete melting prior to OS analysis. A 5 g sample of SFO or melted oleogel, accurately weighed, was used for the analysis.

Afterwards, 50 mL of a solution of acetic acid-isooctane solution (3:2 *v*/*v*) and 0.5 mL of a saturated potassium iodide solution (prepared by dissolving 144 g of potassium iodide in 100 mL of distilled water) were added to the sample. The flask was tightly sealed and shaken manually for 1 min. Then, 30 mL of distilled water, a starch indicator solution (1% *w*/*v*), and 10 drops of lauryl (prepared by dissolving 10 g of lauryl sulfate in 100 mL of distilled water) were added to the mixture. Finally, the sample was titrated with a 0.01 N sodium thiosulfate solution until the brown color disappeared. The peroxide value (*PV*) was calculated according to Equation (3).

(3) $$PV\;\left(\frac{meqO_{2}}{\mathrm{kg}}\right)=\frac{B\times N\times 100\;}{A}$$

where *B* is the volume (mL) of 0.01 N sodium thiosulfate solution required for the titration; *N* is the normality of the sodium thiosulfate solution; and *A* is the amount of sample (g).

### 2.5. Statistical Analysis

The experiments were conducted in triplicate, and the results were expressed as mean ± standard deviation. Statistical comparison between treatments was performed using analysis of variance (ANOVA) followed by Tukey’s test (*p* < 0.05), using the R i386 software (version 3.6.1, R Foundation for Statistical Computing, Vienna, Austria).

## 3. Results and Discussion

### 3.1. CALB Hydrolytic and Transesterification Activities

CALB is a commercially available GRAS lipase that has found wide application in the industrial production of ester compounds and/or hydrolysis of fats and oils [33]. Transesterification in a non-aqueous medium lead to the formation of CE and water; however, the presence of water can impair the transesterification reaction due to the simultaneous occurrence of the hydrolysis reaction [17]. Therefore, it is crucial to evaluate the hydrolytic and transesterification activities of CALB, since the high hydrolytic activity of lipases can represent a significant drawback for CE synthesis. The results showed that the transesterification activity (468.7 ± 6.2 U. g^−1^) measured with free fatty acids was almost 5 times higher than the hydrolytic activity (10.69 ± 0.03 U. g^−1^). Thus, the higher activity exhibited by CALB rendered it a suitable catalyst for CE synthesis.

### 3.2. PB Experimental Design: Selection of Ester Production Conditions

**Figure 1:**
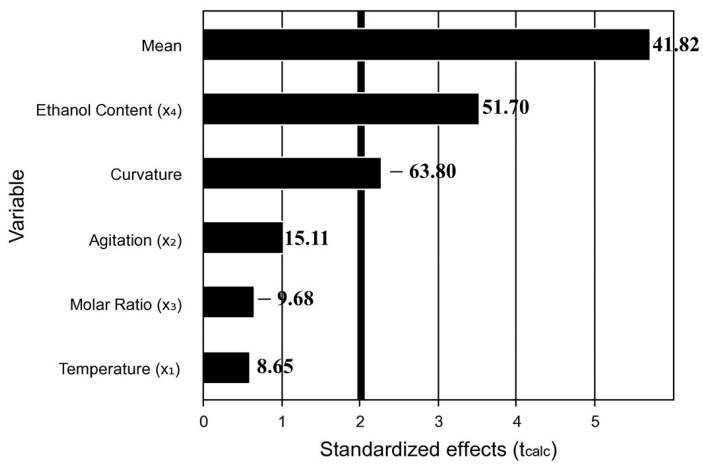
Pareto chart showing the effect of experimental conditions on sorbitan behenate (SB) synthesis.

The production of sorbitol ester catalyzed by CALB was evaluated using the Plackett-Burman statistical design from 11 experimental runs. The PB design was used to identify the main process variables influencing sorbitol consumption. The Plackett–Burman design shows the advantage of screening many factors simultaneously with a very low number of runs but allows for the analysis of only the main significant effects. Table 1 provides the consumption values obtained in the experimental runs, ranging from 3.9% to 95.0%, while the Pareto chart illustrates the impact of the four parameters evaluated (Figure 1). Thus, for validation, triplicate using the conditions of run 1 was performed, obtaining a mean and standard deviation of 95.8% ± 0.3, respectively.

Usually, an excess of substrate can increase the mass transfer rate to the enzyme active site and act by reducing the water activity in the reaction medium. However, increasing sorbitol had the opposite effect. This is probably caused by the high hygroscopicity of sorbitol that removes the hydration layer of the enzyme, essential for maintaining the active conformation of this protein [34]. In turn, increasing temperature and agitation speed had a positive effect on consumption, which may be related to the increased solubility and diffusion of the components in the reaction medium [35]. But, notably, among all the dependent variables studied, only the ethanol content was statistically significant in relation to the consumption rate. This finding can be attributed to the fact that an excess of water hampers the transport of the hydrophobic component within the reaction medium to the vicinity of the enzyme, thereby limiting the overall efficiency of the reaction [21]. Thus, despite the non-significance of some variables, condition PB 1, which used the highest temperature (60 °C) (+1), agitation speed (200 rpm) (−1), water-free reaction medium, and the lowest amount of sorbitol (−1), was selected to produce the oleogelator to be evaluated. Regarding process efficiency, reaction time remains a hurdle for certain enzyme-catalyzed reactions compared to chemical pathways. Nevertheless, in this study, it was demonstrated that CALB promoted satisfactory sorbitol consumption under certain experimental conditions, with a reaction time of 48 h. Due to the transesterification capacity of CALB and the presence of six hydroxy groups capable of accepting hydrophobic groups, a variety of esters can be formed. Although the purification steps are common for carbohydrate esters, in this study, the impurity of the product with molecules of different chains and hydrophilic-lipophilic balance is desirable, aiming at the formation of a multicomponent oleogelator. In addition to the numerous possibilities of combination between hydroxyls and fatty acids subject to esterification, unreacted portions interact in the composition and structuring of the oleogel, forming a material with partial melting and crystallization profiles according to the change in temperature, as can be observed in the calorimetry and temperature scanning results in the subsequent sections.

**Figure 2:**
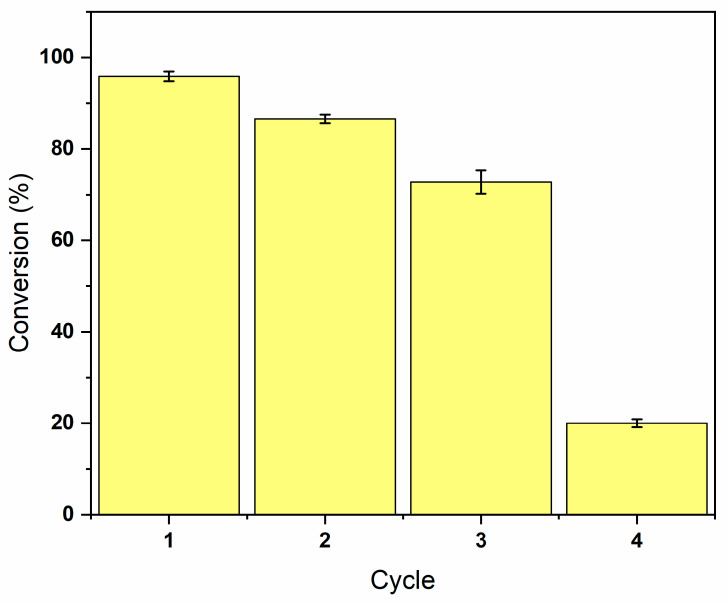
Consumption of sorbitan behenate (SB) according to successive reuse of CALB in the SB synthesis.

In addition, the reuse of enzymes was assessed in order to verify the economic feasibility of large-scale production [36,37]. As illustrated in Figure 2, the reuse of CALB in SB production is feasible for 3 cycles, maintaining sorbitol consumptions above 75%. Consumption decreased to 20% in the fourth cycle, hampering the reuse of the enzyme due to the low efficiency of the process. The gradual decline in enzymatic consumption can be attributed to the loss of enzyme activity caused by the adsorption of alcohols on the enzyme surface [38]. In order to increase the enzyme’s capacity and stability and make this process more industrially viable through a greater number of cycles for the production of the SB structurant, strategies such as the optimization of the reaction medium and modifications in the enzyme immobilization should be the target of study in future studies.

### 3.3. SB Characterization

**Figure 3:**
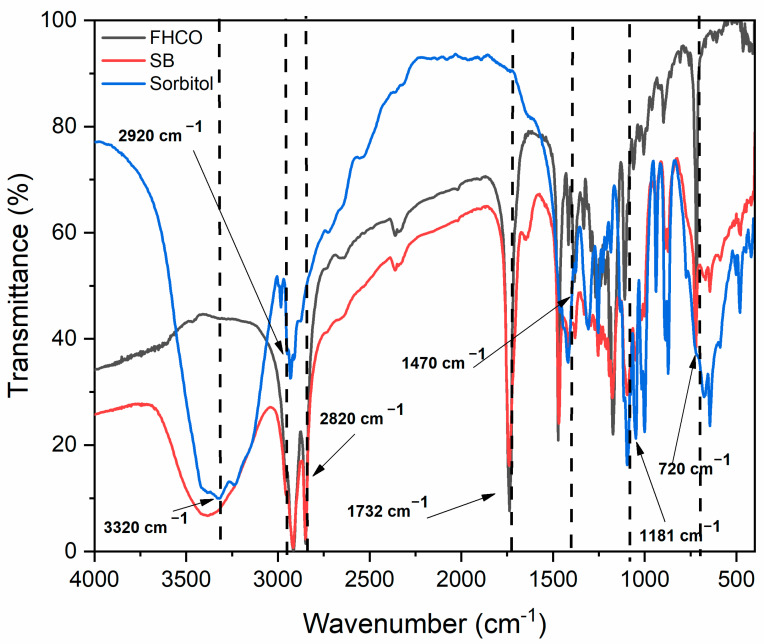
FTIR spectra of sorbitan behenate (SB), sorbitol, and FHCO. Black dotted line black dotted lines indicate highlighted bands.

The FTIR technique is an effective and straightforward way to obtain information about changes in the chemical structure [39]. Herein, the FTIR technique was employed to qualitatively assess the modification in the FHCO structure with the insertion of the hydrophilic moiety (sorbitol). FTIR spectra are presented in Figure 3. Taking into account the typical structural features observed in CEs, it can be noted that the occurrence of FHCO-transesterification was endorsed by the appearance of a spectral band between 3600 and 3100 cm^−1^ (O–H vibration), which could not be detected for FHCO. This structural change in the spectrum from FHCO to SB qualitatively indicates the occurrence of the reaction and the insertion of hydroxyl groups of sorbitol (hydrophilic component) into the fatty acids (behenic or stearic) structure of FHCO. This spectrum, together with indirect monitoring of sorbitol consumption, indicates enzyme action in the production of sorbitol esters. However, part of the peak intensity at this wavelength can also be attributed to unreacted FHCO and sorbitol that can interact physically. Furthermore, six prominent bands were identified: 2920 and 2850 cm^−1^ (corresponding to asymmetric and symmetric stretching of CH_2_ groups), 1732 cm^−1^ (indicative of C=O stretching in the ester bond), 1470 cm^−1^ (representing the bending of CH_2_ groups), 1181 cm^−1^ (attributed to C–O–C stretching in the ester bond), and 720 cm^−1^ (associated with the bending of C=C bonds in alkene compounds) [19,34,40,41,42].

### 3.4. Oleogels Characterization

#### 3.4.1. Rheological Measurements

**Figure 4:**
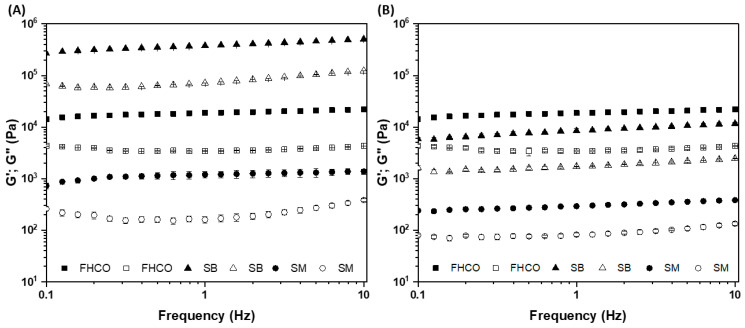
Frequency sweeps for FHCO, SB, and SM oleogels incubated at 5 °C (**A**) and 25 °C (**B**). Solid and open symbols represent elastic (G′) and viscous (G″) moduli.

Rheological measurements were performed at 0.1% strain within the linear viscoelasticity region (identified from strain sweep), in which the sample structure does not show irreversible deformation under applied forces (G′ and G″ are independent of the applied strain). FHCO and SB oleogels showed LVR up to 0.1–0.2% strain at 25 °C, while SM showed an extended LVR at higher strain values (10–100%). Subsequently, frequency sweeps were performed at 5 and 25 °C at 0.1% strain. Figure 4 shows the frequency sweeps of the FHCO, SB, and SM oleogels. All samples exhibited a dominant storage modulus (*G*′) compared to loss modulus (*G*″), indicating a predominance of the elastic (G′) over the viscous (G″) property typical of gel-like behavior.

Surprisingly, the FHCO oleogel exhibited a slight decrease in its elastic property at the lowest temperature in comparison to the highest temperature. This phenomenon can be attributed to the α polymorphism exhibited by longer fatty acid chains, needing a longer time to aggregate into more stable polymorphs. The fast-cooling rate in FHCO oleogel accelerates its crystallization process, resulting in a rapid increase in viscosity. Consequently, this increase in viscosity poses a restriction on mass transfer, preventing the transition from the metastable form to thermodynamically more stable crystalline structures. Moreover, the transition from the crystalline network is significantly hindered due to the restricted molecular mobility in the highly viscous environment of the oleogel. The slow evolution of polymorphic transitions in long-chain fully hydrogenated fatty acids, such as those in FHCO, further exacerbates this effect. Thus, over time, a gradual transformation may enhance the rigidity of the network [16,43].

In fact, the SB oleogel at 5 °C demonstrated a robust structure with the highest G′ values (>10^5^ Pa), standing out in comparison to FHCO and SM. This behavior suggests that the sorbitol head group plays an important role in increasing the strength of the oleogels. Although the FHCO and SB oleogelators share a similar fatty chain structure, additional free hydroxyl terminal groups of the formed esters can potentially provide an additional platform for molecular interactions, such as hydrogen bonds, in the crystal lattice. Such interactions are further favored with decreasing temperature, directly affecting the structure formed of the gel network, as observed in the microscopies in Section 3.4.3 [44]. The highest elastic modulus (G′) of SB oleogels at 5 °C highlights the successful strategy of combining esters with distinct fatty chains, resulting in more structured food formulations under refrigeration than in FHCO-based oleogels (without esterification).

**Figure 5:**
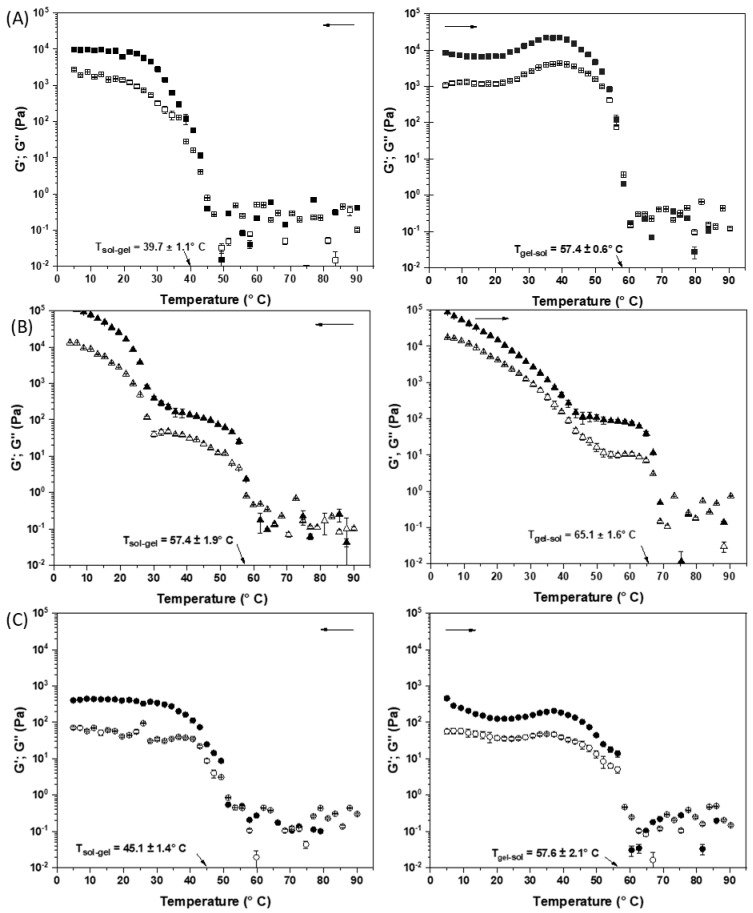
Temperature sweeps of (**A**) FHCO, (**B**) SB, and (**C**) SM oleogels. Cooling (**left**) and heating (**right**) steps. Solid symbol (G′) and open symbol (G″).

An elastic modulus higher than that of the control oleogels is initially the main parameter of interest in the technological properties of oleogels. However, in food applications, the thermorheological response provides insights for the design of reformulated and new products using oleogels to replace saturated fats. In this sense, the robustness of the SB oleogel at 5 °C is relevant for the stability and texture of refrigerated products, but the degree of structuring of the oleogel needs to vary according to the application. For instance, the spreadability of margarines, cream cheeses, or other spreads is associated with a pasty texture (less structuring) at temperatures above 10 °C to room temperature [6]. Or swallowing occurs at human body temperature (37 °C), and melting is relevant, directly impacting sensory perception. Thus, the gelation-melting behavior of the oleogels was investigated by non-isothermal rheological measurements. The cooling and heating profiles of the samples are illustrated in Figure 5. At the beginning of the cooling stage, all samples exhibited a *G*″ value greater than G′, indicating the predominance of viscous behavior until the crossover of the dynamic moduli (*G*3 = G′). A gradual increase in G’ was observed as the temperature decreased, which is related to the organization of the crystals until reaching the construction of the 3D network and formation of the oleogel structure [2]. FHCO and SM oleogels exhibited a sharp transition during the cooling stage at relatively high temperatures (between 40 and 45 °C for FHCO and 45–50 °C for SM). On the other hand, SB showed a 2-step network formation upon cooling (Figure 5B-left), indicating a more complex crystallization profile attributed to the mixed gel network structured by unreacted FHCO and/or by the presence of sorbitol esters (with predominant fatty chains of behenic and stearic acid). Thus, this multi-component blend clearly modulated the behavior of SB oleogels, presenting a gradual melting profile. On the one hand, although the release of oil from the immobilized structure is desired to provide coating and lubrication during shear stress during chewing if it occurs abruptly (as seen in FHCO and SM oleogels), it may result in excessive oiliness. On the other hand, the progressive decay of G′ increasing temperature may favor a more balanced sensation and a more pleasant sensory experience.

During the reheating step, the profiles were similar to the cooling profile, mainly characterized by a sharp drop for FHCO and SM oleogels upon reaching the melting temperature of the crystal lattice. In SB oleogels, two distinct steps are observed before the gel reaches the melting point, indicating the presence of different crystalline species involved in network formation. The complete melting temperature of the SB oleogel was approximately 65 °C, close to the melting temperatures of the predominant fatty acids in the structure, namely stearic acid (69 °C) and behenic acid (72 °C). Above 70 °C, oleogels melt, and highly scattered data are observed. This behavior is a consequence of the sensitivity limitations of the equipment in this low strain range. However, low strain was required to maintain the linear viscoelasticity range at lower temperatures (structured oleogel). Thus, although the data after gel melting indicate only qualitative moduli values, the discussion focusing on gel formation was not impaired.

#### 3.4.2. Thermal Properties (DSC)

**Figure 6:**
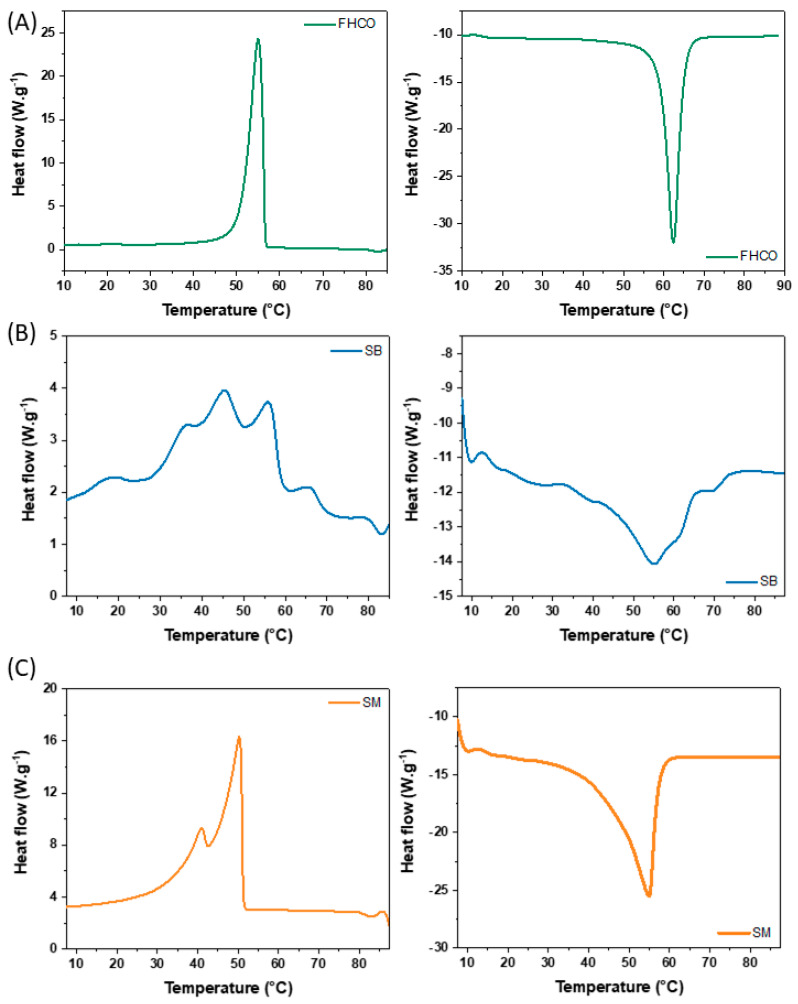
Melting (**left**) and crystallization (**right**) for FHCO (**A**), SB (**B**), and SM (**C**) oleogels.

As mentioned in the previous section, the thermal behavior of oleogels should resemble that of fats to ensure a more accurate imitation of their sensory attributes [45]. Figure 6 shows the thermograms of the FHCO, SB, and SM oleogels. The FHCO oleogel exhibited well-defined peaks, with an onset crystallization temperature around 55 °C and a melting temperature also close to 50 °C (Table S1). The thermograms of the SM oleogel identified an endothermic peak around 55 °C (melting) and two exothermic peaks close to 40 °C and 50 °C (crystallization). For the SB oleogel, different peaks were observed during the cooling and heating steps, which is directly related to the multicomponent nature of the structure. SB oleogel showed a broad melting peak between approximately 20° and 70 °C, a gradual melting behavior that is interesting for sensory attributes during oral processing. The partial melting of SB oleogel at different temperature ranges allows the release of the oil immobilized in the three-dimensional network gradually, which may result in greater lubrication of the oral cavity without excessive oiliness and, therefore, a pleasant sensation during chewing. In the crystallization profile, the SB oleogel exhibited four broad and overlapping peaks also ranging from 20 to 70 °C, which can be associated with the different slopes observed during cooling in the rheological measurements (Figure 5B-left). Although the fatty chains are predominantly C18:0 and C22:0, potentially all of them can be transesterified into a sorbitol backbone. Thus, different species can structure, such as unreacted FHCO and the esters produced by enzymatic transesterification, modifying the mechanism of formation of the oleogel crystal network. Such a combination showed that SB oleogels presented greater sensitivity to temperature variation, in agreement with the rheological results that showed a higher elastic modulus variation with temperature (10^−2^ to 10^5^ Pa) than FHCO oleogels (10^−1^ to 10^4^ Pa).

#### 3.4.3. Polarized Light Microscopy (PLM)

**Figure 7:**
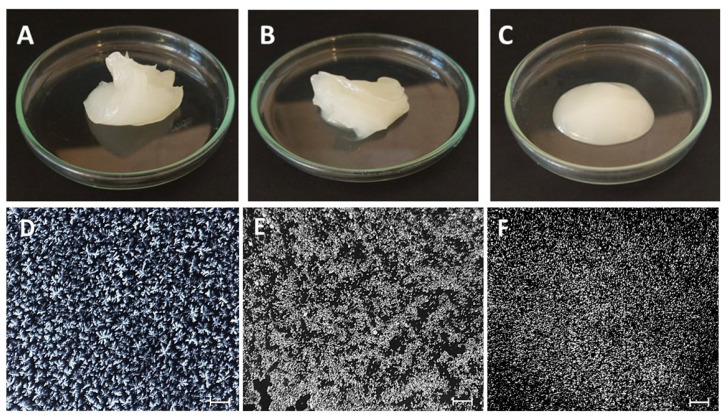
Macroscopic appearance and polarized light micrographs of oleogels. (**A**,**D**)—FHCO oleogel; (**B**,**E**)—SB oleogel; (**C**,**F**)—SM oleogel. Scale: 100 μm.

The crystalline morphology of the oleogel allows an understanding of the characteristics of the structure responsible for trapping the liquid oil. Figure 7 shows the birefringent crystalline structure of the gels, as well as their macroscopic appearance. As previously hypothesized, all samples showed crystallization as the mechanism for the 3D oleogel structure, although it is clear that the type of structuring agent affected the crystal morphology. The SM oleogel (Figure 7F) exhibited loosely packed tiny crystals. Despite the homogeneity, these crystals were not able to form three-dimensional networks capable of immobilizing the oil, according to the visual appearance of this oleogel (Figure 7C). This behavior is in agreement with the findings of [46], in which the concentration of SM used (10% *w*/*w*) was insufficient to form a dense and structured network. A concentration of at least 18% (*w*/*w*) of pure SM is required to form a three-dimensional network [11].

Visually, the FHCO oleogels (Figure 7A) did not differ from the SB oleogels (Figure 7B) at room temperature. However, the FHCO oleogel showed rosette-like crystals (Figure 7D), which were more densely packed and evenly distributed than the SB oleogels. Despite having a similar fatty acid profile to that of FHCO, the SB oleogels (Figure 7E) exhibited crystals more similar to those found in SM oleogels, suggesting that the hydrophilic group has a strong influence on the crystallization mechanism or the possible formation of sorbitan monostearate that governed, at least in part, the crystal formation. It is evident that the prevalence of longer fatty acid chains resulting from the reaction with FHCO led to an increase in crystal size and a denser network structure. Spherulite crystals were observed radiating and branching out, forming a larger and more organized network. Crystal growth appears to occur through the aggregation of smaller units, resulting in the formation of larger structures. This phenomenon explains the greater ability of oleogels to immobilize oil more effectively. Although the SB oleogel network was structured by spherulitic crystals, as well as the SM oleogel, the protection against the formation of hydroperoxides was similar to that presented by the FHCO oleogel, indicating that the degree of structuring was predominant in the ability to block the penetration of oxygen into the structures of the oleogels and was mainly due to the strength of the oleogels [47]. These findings are in agreement with the FD values observed for FHCO, SB, and SM oleogels, which were 1.67 ± 0.02, 1.80 ± 0.03, and 1.66 ± 0.01, respectively. The incorporation of SB into the gel network resulted in a higher FD. Briefly, the presence of unreacted fatty acid chains and sorbitol (modifying the solvent environment), as well as the insertion of the hydrophilic group in the formation of esters, favored the formation of a network with varied structuring depending on the temperature. 

#### 3.4.4. Oxidative Stability (OS)

**Figure 8:**
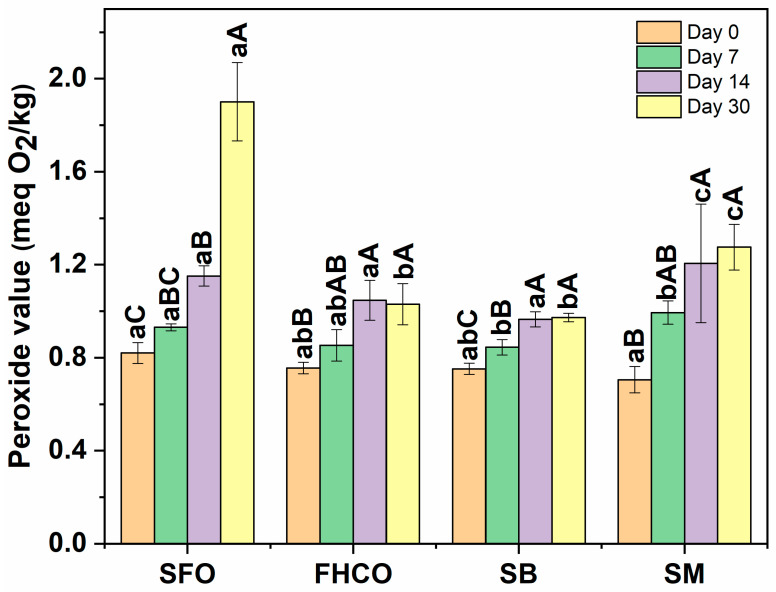
Peroxide value of SFO (control) and FHCO, SB, and SM oleogels after 0-, 7-, 15-, and 30-day(s) of storage at 25 °C. Different lowercase letters mean statistical difference between all samples for each storage time. Different capital letters mean statistical difference between the same sample for different storage times.

Oxidative stability data of SFO and oleogels are available in Figure 8. Oxidative stability was monitored by peroxide value (PV), which indicates the amount of primary products formed during lipid oxidation [48]. Before evaluating the PV of the control sample (SFO) and oleogels (all subjected to the same thermal process conditions), the PV of sunflower oil without any heating or addition of gelator was measured (0.81 ± 0.06 meqO_2_/kg). This result was similar to the values obtained for the control sample (heated SFO), showing that at least in the initial time, heating did not affect the formation of peroxides. After preparation, oleogels and control samples were stored at refrigerated temperature for 48 h before performing the first PV measurement (day 0 of storage). The effect of the three-dimensional network formed in the oleogels was observed at day 30, as the PV value of SFO was significantly (*p* < 0.05) higher than that of all oleogels. This difference is associated with the formation of networks in oleogels that play a role as a physical barrier to protect the organic solvent from the effects of light and oxygen during storage, thereby increasing their stability compared to liquid oil [49].

#### 3.4.5. Oil Binding Capacity (OBC)

OBC is a crucial feature of oleogels, indicating the extent to which liquid oil is trapped within the three-dimensional network of gelators [45]. Ensuring the physical stability of oleogels is of utmost importance to enable their application in commercial products, due to their high liquid oil content (90% *w*/*w*) [16]. Table 2 presents the OBC results at temperatures of 5 and 25 °C, revealing a range of OBC values between 40 and 88%. Notably, SM oleogels demonstrated the lowest OBC values at both 5 and 25 °C. Although they have the potential to enhance the structuring capacity of other gelators, SM gelators alone have limited effectiveness in oleogel formation [50]. In contrast, FHCO oleogels displayed the highest OBC values at 25 °C, while SB oleogels exhibited the highest values at 5 °C. This finding is in line with results observed on the elastic properties of oleogels at different temperatures. This behavior is attributed to the long fatty acid’ chains present in FHCO aggregated in more stable forms at 25 °C due to the rapid aggregation that restricts the mass transfer that occurs at 5 °C [16,43]. Both SM and SB oleogels exhibited a statistically significant increase in OBC values with decreasing temperature, whereas FHCO oleogels did not exhibit such variation. The higher OBC value of SB oleogel at 5 °C can be attributed to the dense network formed by small crystals (Figure 7E), effectively reducing the presence of pores and expanding the surface area available for oil holding [51]. However, despite the reduction of OBC for SB oleogels with increasing temperature, this “melting” is expected considering applications as a cooking and spreading ingredient.

## 4. Conclusions

This study reinforces the role of rheological response to temperature in the development of oleogels as technically feasible and sensorially acceptable replacements for saturated fats. Carbohydrate-based esters, such as the mixture produced in this study and used as oleogelators, are considered biocompatible and safe for consumption, standing out as ingredients with significant potential for use as oleogelators in food systems. The multicomponent mixture of enzymatically synthesized ultra-long-chain esters proved to be a promising approach to overcome the typical limitations of conventional and purified oleogelators, enabling the formation of oleogels with distinct structural/thermal properties. SB oleogels were notable for their enhanced structural robustness under refrigeration (5 °C). However, thermal analyses and non-isothermal oscillatory rheology revealed a wide temperature range melting profile of SB oleogels—a critical attribute for food applications that require stability during storage and a more pasty texture under processing and consumption conditions. In addition, by inhibiting the formation of hydroperoxides and preserving the oxidative stability of sunflower oil over 30 days of storage, the developed oleogels demonstrate advantages not only in terms of replacing saturated fats but also in food preservation and safety. Therefore, our findings open new perspectives for the use of ultra-long-chain combined with other esters in the design of multifunctional oleogels, aligned with industrial and consumer demands for healthier and more sustainable solutions. Future studies should deepen the understanding of the molecular mechanisms that govern the interactions between the various oleogel components and explore practical applications in complex food matrices, further increasing the impact of these systems in replacing saturated fats.

## Figures and Tables

**Table 1:** Independent variables and consumption of sorbitol esters using conditions defined by the PB experimental design.

Essay	Temperature (°C)	Rotational Velocity (rpm)	FHCO:Sorbitol Molar Ratio	Ethanol Content (%)	Sorbitol Consumption (%)
1	60	200	1:1	100	95.8
2	60	500	1:1	50	30.8
3	60	500	1:5	50	24.9
4	40	500	1:5	100	85.3
5	60	200	1:5	100	33.8
6	40	500	1:1	100	56.5
7	40	200	1:5	50	3.9
8	40	200	1:1	50	4.3
9	50	350	1:3	75	9.9
10	50	350	1:3	75	9.3
11	50	350	1:3	75	8.4

**Table 2:** Oil binding capacity of SB, SM, and FHCO oleogels.

Sample	OBC (%)	
	5 °C	25 °C
SB	88.4 ± 3.1 ^Aa^	68.6 ± 1.8 ^Bb^
SM	62.4 ± 4.4 ^Ac^	40.1 ± 2.0 ^Bc^
FHCO	76.6 ± 1.4 ^Ab^	77.5 ± 0.8 ^Aa^

SB: sorbitan behenate oleogel; SM: sorbitan monostearate oleogel; FHCO: fully hydrogenated crambe oil oleogel. Different lowercase letters in the same column mean statistical difference between the samples. Different capital letters mean statistical differences for each sample between the different storage temperatures.

## Data Availability

The original contributions presented in this study are included in the article/Supplementary Materials. Further inquiries can be directed to the corresponding author.

## Supplementary Materials

The following supporting information can be downloaded at: https://www.mdpi.com/article/10.3390/foods14061030/s1, Table S1: Crystallization temperature (T_C1, onset_), melting (ΔH_m_) and crystallization (ΔH_c_) enthalpy from DSC measurements.

## References

[1] Davidovich-Pinhas M. (2018). Oleogels. Polymeric Gels.

[2] Martins A.J., Cerqueira M.A., Fasolin L.H., Cunha R.L., Vicente A.A. (2016). Beeswax organogels: Influence of gelator concentration and oil type in the gelation process. Food Res. Int..

[3] Maki K.C., Dicklin M.R., Kirkpatrick C.F. (2021). Saturated fats and cardiovascular health: Current evidence and controversies. J. Clin. Lipidol..

[4] Liu L., Gao Z., Chen G., Yao J., Zhang X., Qiu X., Liu L. (2024). A comprehensive review: Impact of oleogel application on food texture and sensory properties. Food Sci. Nutr..

[5] Singh A., Auzanneau F.-I., Rogers M.A. (2017). Advances in edible oleogel technologies—A decade in review. Food Res. Int..

[6] Bayarri S., Carbonell I., Costell E. (2012). Viscoelasticity and texture of spreadable cheeses with different fat contents at refrigeration and room temperatures. J. Dairy Sci..

[7] Liu L., Abdullah, Tian W., Chen M., Huang Y., Xiao J. (2023). Oral sensation and gastrointestinal digestive profiles of bigels tuned by the mass ratio of konjac glucomannan to gelatin in the binary hydrogel matrix. Carbohydr. Polym..

[8] Ji L., Gravelle A.J. (2025). Modulating the behavior of ethyl cellulose-based oleogels: The impact food-grade amphiphilic small molecules on structural, mechanical, and rheological properties. Food Hydrocoll..

[9] Airoldi R., da Silva T.L.T., Ract J.N.R., Foguel A., Colleran H.L., Ibrahim S.A., da Silva R.C. (2022). Potential use of carnauba wax oleogel to replace saturated fat in ice cream. J. Am. Oil Chem. Soc..

[10] Sagiri S.S., Samateh M., John G. (2024). Investigating the Emulsifying Mechanism of Stereoisomeric Sugar Fatty Acyl Molecular Gelators. Langmuir.

[11] Swe M.T.H., Asavapichayont P. (2018). Effect of silicone oil on the microstructure, gelation and rheological properties of sorbitan monostearate–sesame oil oleogels. Asian J. Pharm. Sci..

[12] Daniel J., Rajasekharan R. (2003). Organogelation of plant oils and hydrocarbons by long-chain saturated FA, fatty alcohols, wax esters, and dicarboxylic acids. J. Am. Oil Chem. Soc..

[13] de Oliveira G.M., Badan Ribeiro A.P., dos Santos A.O., Cardoso L.P., Kieckbusch T.G. (2015). Hard fats as additives in palm oil and its relationships to crystallization process and polymorphism. LWT—Food Sci. Technol..

[14] Moreira D.K.T., Ract J.N.R., Ribeiro A.P.B., Macedo G.A. (2017). Production and characterization of structured lipids with antiobesity potential and as a source of essential fatty acids. Food Res. Int..

[15] Moreira D.K.T., Santos P.S., Gambero A., Macedo G.A. (2017). Evaluation of structured lipids with behenic acid in the prevention of obesity. Food Res. Int..

[16] Callau M., Sow-Kébé K., Nicolas-Morgantini L., Fameau A.-L. (2020). Effect of the ratio between behenyl alcohol and behenic acid on the oleogel properties. J. Colloid Interface Sci..

[17] Nguyen P.C., Nguyen M.T.T., Kim J.-H., Hong S.-T., Kim H.-L., Park J.-T. (2021). A novel maltoheptaose-based sugar ester having excellent emulsifying properties and optimization of its lipase-catalyzed synthesis. Food Chem..

[18] Teng Y., Xu Y. (2007). A modified para-nitrophenyl palmitate assay for lipase synthetic activity determination in organic solvent. Anal. Biochem..

[19] Arniza M.Z., Hoong S.S., Yusop M.R., Hayes D.G., Yeong S.K., NSMariam N.M. (2020). Regioselective Synthesis of Palm-Based Sorbitol Esters as Biobased Surfactant by Lipase from Thermomyces lanuginosus in Nonaqueous Media. J. Surfactants Deterg..

[20] Neta N.S., Teixeira J.A., Rodrigues L.R. (2015). Sugar Ester Surfactants: Enzymatic Synthesis and Applications in Food Industry. Crit. Rev. Food Sci. Nutr..

[21] Pyo S.-H., Chen J., Ye R., Hayes D.G. (2019). Sugar Esters. Biobased Surfactants.

[22] Teng Y., Stewart S.G., Hai Y.W., Li X., Banwell M.G., La P. (2021). Sucrose fatty acid esters: Synthesis, emulsifying capacities, biological activities and structure-property profiles. Crit. Rev. Food Sci. Nutr..

[23] Zago E., Joly N., Chaveriat L., Lequart V., Martin P. (2021). Enzymatic synthesis of amphiphilic carbohydrate esters: Influence of physicochemical and biochemical parameters. Biotechnol. Rep..

[24] de Medeiros L.L., da Silva F.L.H., de Queiroz A.L.M., de Oliveira Y.S.L., de Souza Junior E.F., Madruga M.S., da Conceição M.M. (2020). Structural-chemical characterization and potential of sisal bagasse for the production of polyols of industrial interest. Braz. J. Chem. Eng..

[25] Czaikoski A., da Cunha R.L., Menegalli F.C. (2020). Rheological behavior of cellulose nanofibers from cassava peel obtained by combination of chemical and physical processes. Carbohydr. Polym..

[26] Barroso N.G., Okuro P.K., Ribeiro A.P.B., Cunha R.L. (2020). Tailoring Properties of Mixed-Component Oleogels: Wax and Monoglyceride Interactions Towards Flaxseed Oil Structuring. Gels.

[27] Picone C.S.F., Cunha R.L. (2011). Influence of pH on formation and properties of gellan gels. Carbohydr. Polym..

[28] Okuro P.K., Malfatti-Gasperini A.A., Vicente A.A., Cunha R.L. (2018). Lecithin and phytosterols-based mixtures as hybrid structuring agents in different organic phases. Food Res. Int..

[29] Okuro P.K., Tavernier I., Bin Sintang M.D., Skirtach A.G., Vicente A.A., Dewettinck K., Cunha R.L. (2018). Synergistic interactions between lecithin and fruit wax in oleogel formation. Food Funct..

[30] Blake A.I., Marangoni A.G. (2015). The Use of Cooling Rate to Engineer the Microstructure and Oil Binding Capacity of Wax Crystal Networks. Food Biophys..

[31] Martins A.J., Cerqueira M.A., Cunha R.L., Vicente A.A. (2017). Fortified beeswax oleogels: Effect of β-carotene on the gel structure and oxidative stability. Food Funct..

[32] AOCS (2003). Official Method Cd 8-53.

[33] Lee K.P., Kim H.K. (2016). Antibacterial Effect of Fructose Laurate Synthesized by Candida antarctica B Lipase-Mediated Transesterification. J. Microbiol. Biotechnol..

[34] Abdulmalek E., Hamidon N.F., Abdul Rahman M.B. (2016). Optimization and characterization of lipase catalysed synthesis of xylose caproate ester in organic solvents. J. Mol. Catal. B Enzym..

[35] Arcens D., Grau E., Grelier S., Cramail H., Peruch F. (2020). Impact of Fatty Acid Structure on CALB-Catalyzed Esterification of Glucose. Eur. J. Lipid Sci. Technol..

[36] Di X., Zhang Y., Fu J., Yu Q., Wang Z., Yuan Z. (2019). Biocatalytic upgrading of levulinic acid to methyl levulinate in green solvents. Process Biochem..

[37] Zhang Y., Di X., Wang W., Song M., Yu Q., Wang Z., Yuan Z., Chen X., Xu H., Guo Y. (2020). Kinetic study of lipase-catalyzed esterification of furoic acid to methyl-2-furoate. Biochem. Eng. J..

[38] Mulalee S., Srisuwan P., Phisalaphong M. (2015). Influences of operating conditions on biocatalytic activity and reusability of Novozym 435 for esterification of free fatty acids with short-chain alcohols: A case study of palm fatty acid distillate. Chin. J. Chem. Eng..

[39] Haafiz M.K.M., Hassan A., Zakaria Z., Inuwa I.M. (2014). Isolation and characterization of cellulose nanowhiskers from oil palm biomass microcrystalline cellulose. Carbohydr. Polym..

[40] Ji S., Jia C., Cao D., Li S., Zhang X. (2020). Direct and selective enzymatic synthesis of trehalose unsaturated fatty acid diesters and evaluation of foaming and emulsifying properties. Enzyme Microb. Technol..

[41] Tsupko P. (2020). Sugar-Derived Oleogels in Cosmetics and Food Applications: A Case Study of Trehalose and Mannitol-Based Gelators. City University of New York (CUNY). https://academicworks.cuny.edu/cc_etds_theses/823.

[42] Wei W., Feng F., Perez B., Dong M., Guo Z. (2015). Biocatalytic synthesis of ultra-long-chain fatty acid sugar alcohol monoesters. Green Chem..

[43] Kim G.Y., Marangoni A.G. (2017). Crystallization Behavior of High Behenic Acid Stabilizers in Liquid Oil. J. Am. Oil Chem. Soc..

[44] Rosen-Kligvasser J., Davidovich-Pinhas M. (2021). The role of hydrogen bonds in TAG derivative-based oleogel structure and properties. Food Chem..

[45] Thakur D., Singh A., Prabhakar P.K., Meghwal M., Upadhyay A. (2022). Optimization and characterization of soybean oil-carnauba wax oleogel. LWT—Food Sci. Technol..

[46] Godoi K.R.R.D., Basso R.C., Ming C.C., da Silva A.Á., Cardoso L.P., Ribeiro A.P.B. (2020). Crystallization, microstructure and polymorphic properties of soybean oil organogels in a hybrid structuring system. Food Res. Int..

[47] Acevedo N.C., Marangoni A.G. (2010). Characterization of the Nanoscale in Triacylglycerol Crystal Networks. Cryst. Growth Des..

[48] Mohanan A., Nickerson M.T., Ghosh S. (2018). Oxidative stability of flaxseed oil: Effect of hydrophilic, hydrophobic and intermediate polarity antioxidants. Food Chem..

[49] Zeng L., Lin X., Li P., Liu F.-Q., Guo H., Li W.-H. (2021). Recent advances of organogels: From fabrications and functions to applications. Prog. Org. Coat..

[50] Keskin Uslu E., Yılmaz E. (2021). Preparation and characterization of glycerol monostearate and polyglycerol stearate oleogels with selected amphiphiles. Food Struct..

[51] Ahmadi P., Tabibiazar M., Roufegarinejad L., Babazadeh A. (2020). Development of behenic acid-ethyl cellulose oleogel stabilized Pickering emulsions as low calorie fat replacer. Int. J. Biol. Macromol..

