# Supplementary material for: Ultra-Long-Chain Sorbitol Esters Tailoring Thermo-Responsive Rheological Properties of Oleogels
Source: Foods. 2025 Mar 18;14(6):1030. doi: 10.3390/foods14061030 (PMC11941445; doi:10.3390/foods14061030)
Supplement: Supplementary file 1 [file foods-14-01030-s001.zip › foods-3479993-supplementary.pdf]

Supplementary Material

Table S1 - Crystallization temperature ($T_{C1\ onset}$), melting (ΔH_m) and crystallization (ΔH_c) enthalpy from DSC measurements.

Sample	ΔH_m (J/g)	ΔH_c (J/g)	$T_{C1,\ onset}$ (°C)
**FHCO**	21.63 ± 0.88^a	22.85 ± 1.27^a	47.52 ± 0.17a
**SB**	8.35 ± 0.27^b	6.97 ± 0.21^b	40.41 ± 0.25^b
**SM**	2.773 ± 1.03^c	1.046 ± 1.21^c	47.04 ± 1.07^a

Different lowercase letters in the same column mean statistical difference between all samples.
